# Esophageal Hematoma Following Transcatheter Edge-to-Edge Repair of the Mitral Valve: A Rare Complication of Transesophageal Echocardiography

**DOI:** 10.14309/crj.0000000000001822

**Published:** 2025-09-08

**Authors:** Ahmed Aref, Judy Sheffeh, Mohammad Ali Sheffeh, Nishant Aggarwal, Fady Banno, Mohammed Alkhero, Ketan Rana

**Affiliations:** 1 Department of Internal Medicine, Corewell Health William Beaumont University Hospital, Royal Oak, MI; 2 Department of Internal Medicine, Henry Ford Warren, Michigan State University Hospital, Warren, MI; 3 Section of Gastroenterology and Hepatology, Corewell Health William Beaumont University Hospital, Royal Oak, MI

**Keywords:** transesophageal echocardiography, esophageal hematoma, TEER, gastrointestinal bleeding

## Abstract

Transesophageal echocardiography is commonly used to guide structural cardiac interventions but carries a risk of esophageal injury. We present a 79-year-old woman who underwent a Transcatheter Edge-to-Edge Repair of the mitral valve and developed an esophageal hematoma. Clinical course was complicated by intractable gastrointestinal bleeding and sepsis due to acute cholecystitis. She did not survive despite aggressive measures. Our case demonstrates the potential severity of transesophageal echocardiography–related complications, especially in patients with predisposing factors such as esophageal diverticula and anticoagulation use.

## INTRODUCTION

Since its introduction in the 1980s, transesophageal echocardiography (TEE) has been frequently employed in cardiac surgery and interventional cardiology.^1^ Despite extensive use, there are scarce data regarding its safety in structural heart interventions.^2,3^ Transcatheter Edge-to-Edge Repair (TEER) is a minimally invasive procedure performed under TEE guidance to approximate mitral valve leaflets and reduce regurgitation.^4^ We present a case of a patient who underwent TEER complicated by an esophageal hematoma and a challenging clinical course.

## CASE REPORT

A 79-year-old woman with a medical history of severe mitral regurgitation and severe pulmonary hypertension underwent an elective TEER of the mitral valve under general anesthesia (GA). The procedure was uneventful, but she developed postoperative atrial fibrillation requiring initiation of apixaban. She was observed for 2 days and then discharged.

**Figure 1.**
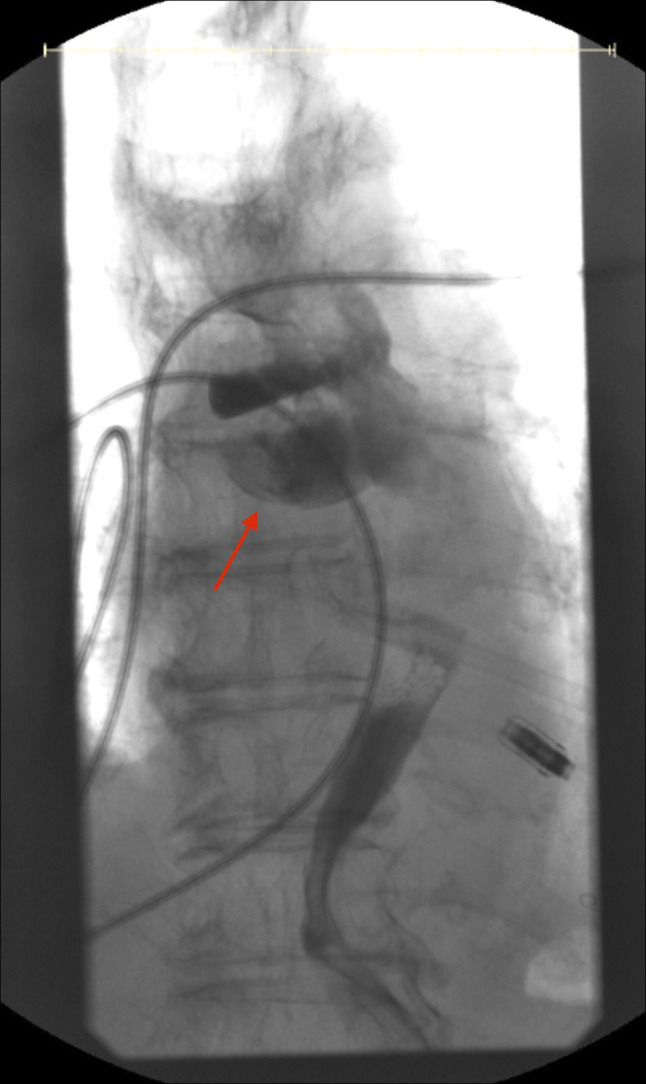
Esophagram showing patulous esophagus and esophageal diverticulum (red arrow).

**Figure 2.**
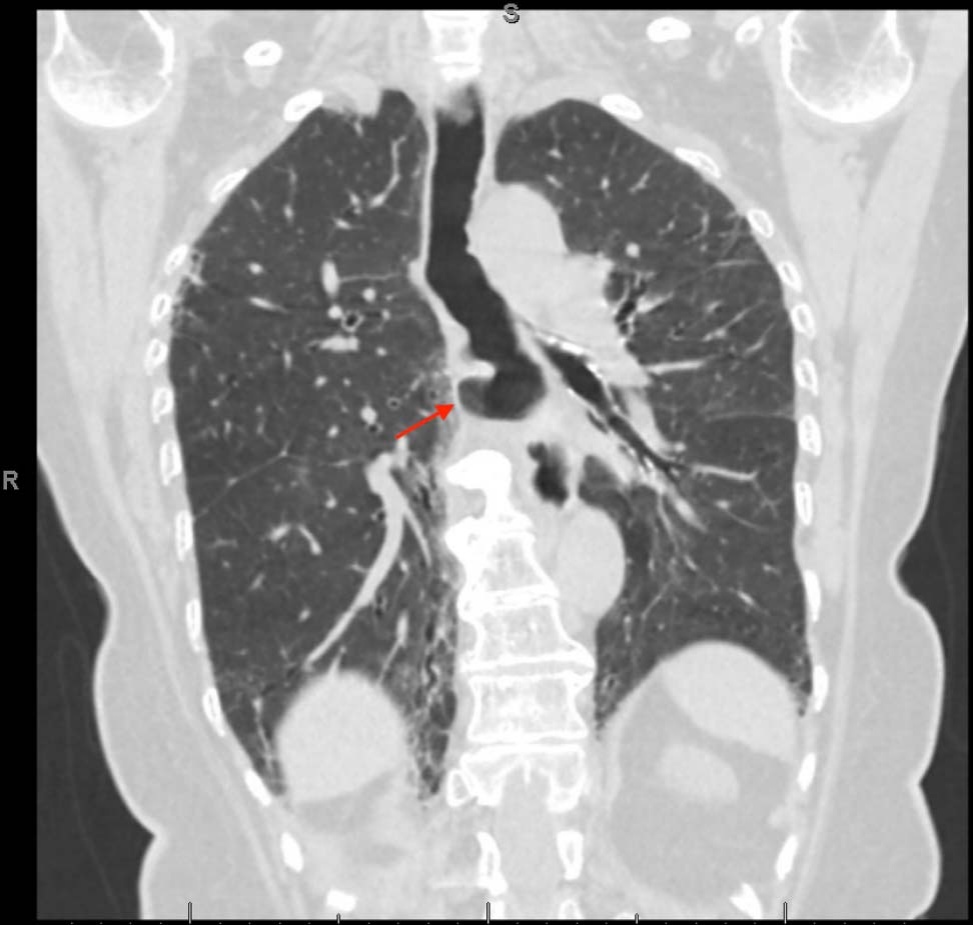
Computed tomography scan 2 months before presentation showing esophageal diverticulum (red arrow).

**Figure 3.**
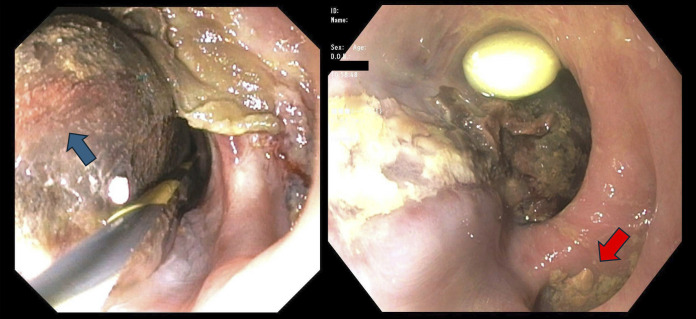
First esophagogastroduodenoscopy showing hematoma (blue arrow) and esophageal diverticulum above hematoma (red arrow).

She had no history of esophageal symptoms or achalasia before the procedure. Mild chest discomfort began immediately after TEER and progressively worsened, leading to emergency department (ED) presentation 2 days post discharge with dysphagia, chest pain, and regurgitation. Aspirin was held, apixaban was switched to intravenous (IV) heparin, and she was given IV pantoprazole 40 mg. An esophagram revealed a patulous esophagus and an esophageal diverticulum (Figure 1) near the gastroesophageal junction, resembling findings on a computed tomography (CT) scan 2 months earlier (Figure 2), although the diverticulum had not been reported at that time. She had no prior endoscopic evaluation. An initial esophagogastroduodenoscopy (EGD) revealed a 5-cm tear in the middle esophagus with an overlying hematoma obstructing the lumen (Figure 3) and 2 diverticula near the hematoma. A pediatric gastroscope was successfully passed, revealing a normal esophagus beyond the hematoma. She was able to advance diet postprocedure. Aspirin was restarted 4 days after the EGD. She was discharged and restarted apixaban 5 days after due to high stroke risk as recommended by cardiology. CT scan was not performed this admission.

**Figure 4.**
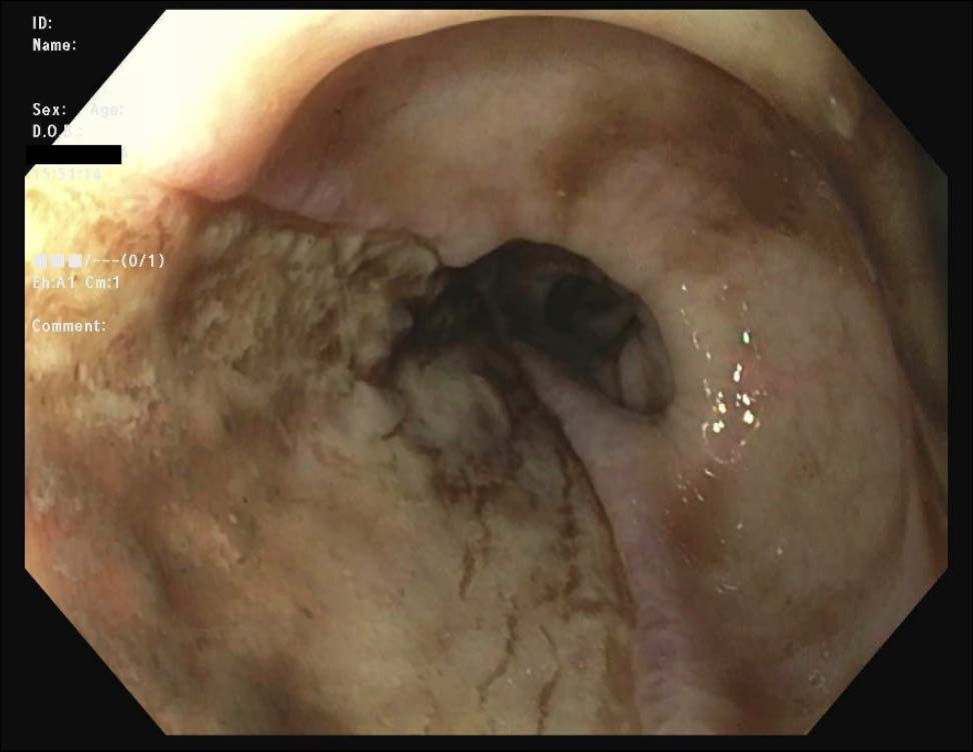
Second esophagogastroduodenoscopy showing esophageal ulcer resulting from ruptured hematoma.

One week later, she returned to the ED with hematemesis and melena. Vital signs were stable, but hemoglobin dropped from 13 g/dL to 9 g/dL. A CT angiography of the thorax showed no active bleeding but showed a distended gallbladder. Again, oral intake and anticoagulation were held. IV pantoprazole was initiated. Abdominal ultrasound confirmed acute cholecystitis. This was managed with a cholecystostomy and antibiotics due to medical instability. A second EGD revealed a clean based 6-cm esophageal ulcer (Forrest III), resulting from ruptured hematoma (Figure 4). Gradually after, her diet was slowly advanced.

**Figure 5.**
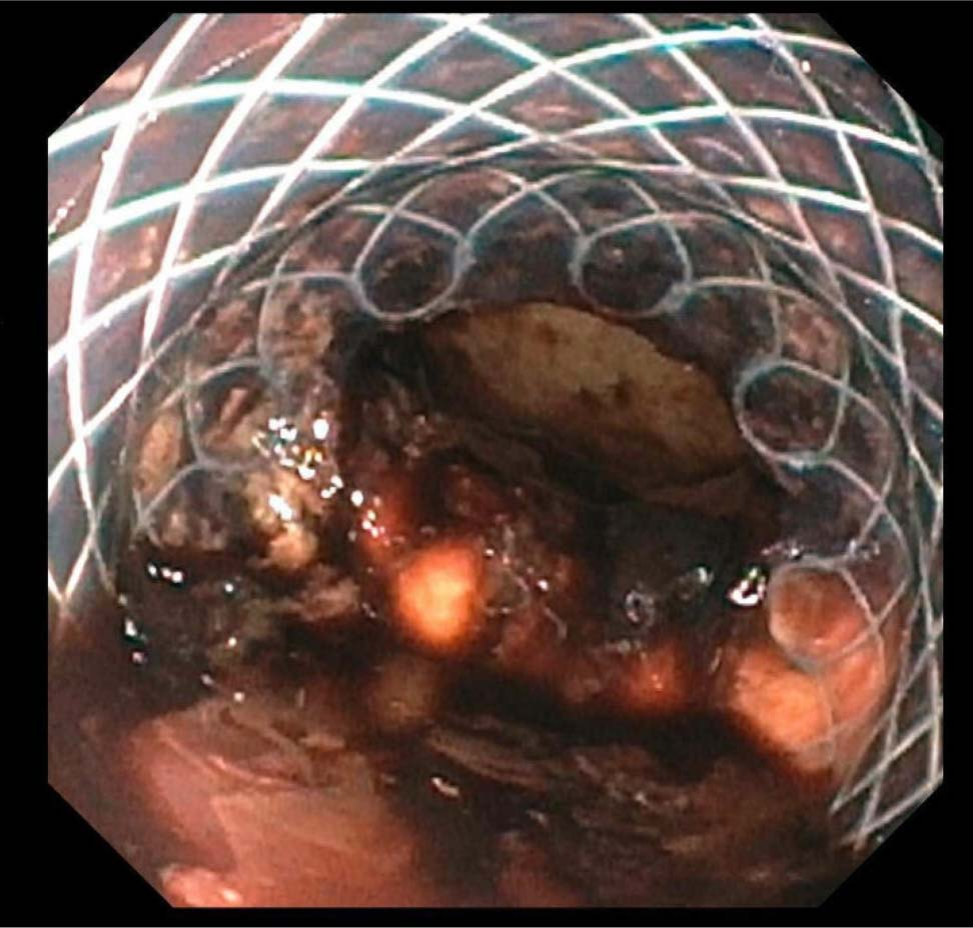
Third esophagogastroduodenoscopy showing esophageal ulcer oozing blood after placement of metal stent.

Unfortunately, she continued to experience persistent hematemesis and melena. A third EGD revealed the previously seen ulcer oozing blood (Forrest IB). No exact source of bleeding was identified, and a fully covered self-expanding metal stent was placed (Figure 5). She remained intubated and was transferred to the intensive care unit. Despite these measures, she continued to experience intractable bleeding. A repeat CT angiography of the thorax showed no active bleeding or evidence of an aorto-enteric fistula. Cardiothoracic surgery recommended supportive measures.

She subsequently developed septic shock necessitating vasopressor support. Despite aggressive management, she continued to deteriorate. After discussing goals of care with her family, compassionate extubation was performed, and she passed away soon after. Blood cultures drawn before her passing grew Enterococcus faecium.

## DISCUSSION

TEE-associated esophageal injuries are likely underreported as not all patients are symptomatic. A prospective analysis conducted by Freitas-Ferraz et al on 50 patients undergoing TEE-guided percutaneous structural heart interventions showed that 43 patients (86%) developed some form of esophageal injury.^3^

Anatomical risk factors for esophageal injury include esophageal ulcers, strictures, diverticula, radiation-induced fibrosis, varices, and extrinsic esophageal compression from enlarged left atrium.^5^ Other risk factors include older age, low body mass index, use of anticoagulants and/or antiplatelet, and longer procedure time.^3,6^ Risk is also higher with GA when compared with local anesthesia. Unconscious patients under GA are unable to cooperate with swallowing and cannot indicate when they are in pain or discomfort, which could lead to mechanical injury during probe insertion.^3^ In a prospective series by Kumar et al, there was a higher risk in women compared with men.^7^ Predisposing risk factors for our patient were age, sex, and undergoing GA. Manipulation of the TEE probe in the presence of anatomical abnormalities likely caused mucosal injury, which, combined with apixaban use, led to hematoma formation.

Common signs and symptoms of esophageal hematoma include chest pain, dysphagia, odynophagia, regurgitation, and gastrointestinal bleeding.^2,8^ Esophageal hematomas due to TEE injury usually present within 12 hours. Atrio-esophageal fistula usually presents 3 to 12 days later with symptoms of sepsis, mediastinitis, or gastrointestinal exsanguination.^7^

Contrast-enhanced CT is typically the first imaging modality to diagnose esophageal hematoma and exclude atrioesophageal fistula before endoscopy.^8^ The structural and functional damage can be assessed further by endoscopy or barium swallow studies.^8,9^ On endoscopy, the hematoma appears as a bluish swelling with or without an overlying tear.^10^ Biopsy should be avoided because of bleeding risk.^9^

In hemodynamically stable patients, esophageal hematomas are typically managed conservatively by holding oral intake and advancing diet as tolerated.^10,11^ Monitoring clinical progress and hematoma resolution can be achieved with serial CT scans and/or contrast swallow studies.^10^ The use of IV acid suppressants until oral route is re-established is rational to avoid acid mediated esophageal injury.^12^ Antibiotics are unlikely to be useful if there are no sign of infection or perforation.^7,9^ A multidisciplinary approach involving gastroenterologists cardiologists and cardiothoracic surgeons is substantial on deciding to stop and resume anticoagulation.^8^ Most cases of esophageal hematomas resolve within 2 weeks.^12^ If patients develop massive bleeding or progressive hematoma expansion, angiography with trans-arterial embolization may be an option; however, no bleeding vessel was identified on our patient's CT scan.^10,13^ Surgery may be required for hemodynamic instability, atrioesophageal fistula, or perforation but is generally associated with poor outcomes.^10^ Esophageal stent placement is a well-established modality for refractory variceal bleeding; however, it was not able to control the bleeding in our case. The presumed cause of death was septic shock due to acute cholecystitis.

## DISCLOSURES

Author contributions: A. Aref: Data curation, review of literature and writing. J. Sheffeh: Writing draft and literature review. M. Sheffeh: Writing draft and literature review. N. Aggarwal: Review and editing. F. Banno: Literature review, editing. M. Alkhero: Review and editing. K. Rana: Reviewed final manuscript and approved for submission and is the article guarantor.

Financial disclosure: None to report.

Informed consent was obtained for this case report.

## ABBREVIATIONS:

CT: computed tomography
ED: emergency department
EGD: esophagogastroduodenoscopy
GA: general anasthesia
IV: intravenous
TEE: transesophageal echocardiography
TEER: transcatheter edge-to-edge repair
